# Performance of the BD FACSPresto near to patient analyzer in comparison with representative conventional CD4 instruments in Cameroon

**DOI:** 10.1186/s12981-020-00309-9

**Published:** 2020-08-17

**Authors:** Bertrand Sagnia, Fabrice Mbakop Ghomsi, Ana Gutierrez, Samuel Sosso, Rachel Kamgaing, Aubin Joseph Nanfack, Nadesh Nji, Georgia Ambada, Abel Lissom, Thibaut Flaurant Tchouangueu, Loveline Ngu Ndengkoh, Irenée Domkam, Godwin Nchinda, Alexis Ndjolo

**Affiliations:** 1 Chantal BIYA International Reference Centre for Prevention and Management of HIV/AIDS (CIRCB) Cameroon, Yaounde, Cameroon; 2 Centre de Sante Catholique de NKOLODOM, Yaounde, Cameroon; 3 Centre de Sante Catholique de BIKOP, Yaounde, Cameroon; 4 Faculty of Sciences, University of Yaounde 1, Yaounde, Cameroon; 5 Faculty of Sciences, University of Dschang, Dschang, Cameroon

**Keywords:** HIV, CD4 T lymphocyte, BD FACSPresto, PIMA POC, FACSCalibur, FACSCount

## Abstract

**Background:**

In the context of scaling the viral load in resource limited settings, following HIV infected patient’s adults and children with CD4+ T-lymphocyte count still very important in settings where the decentralization of treatment still has some challenges. Effective HIV monitoring in these resource-constrained settings needs affordable and reliable CD4+ T lymphocytes enumeration methods. We investigated the validity of a BD FACSPresto POC which is a dedicated system for enumeration that uses immunofluorescent technologies. In this study, we have assessed the sensitivity, specificity and correlation between most representative flow cytometry instruments present in Cameroon with more than 5000 CD4 T cells tests per year including FACSCalibur, FACSCount, and PIMA POC from Becton–Dickinson and ALERE respectively.

**Methods:**

268 patients aged from 1 to 72 years old were enrolled and included in the study after inform consent. The BD FACSPresto POC CD4+ T cell technology was placed at CIRCB and operated by technician staff. HIV infected patients were from Chantal BIYA international reference Center (CIRCB), Centre de Sante Catholique de NKOLODOM, Centre de Sante Catholique de BIKOP and CASS de Nkolndongo—Yaounde We compared the accuracy of the BD FACSPresto and three existing reference technologies with more than 5000 tests per year like FACSCalibur, FACSCount and PIMA according to the number of CD4 test done per year and their repartition in the country. Bland–Altman method and correlation analysis were used to estimate mean bias and 95% limits of agreement and to compare the methods, including analysis by subgroup of participant gestational age. In addition sensitivity and specificity were determined. Statistical significance was set at P-value < 0.05.

**Results:**

The BD FACSPresto POC system has excellent precision, accuracy and linearity for CD4+ T lymphocytes enumeration. Good correlations were obtained between the BD FACSPresto poc system and other single platform methods. Bland–Altman plots showed interchangeability between two machines mean bias BD-FACSPresto vs PIMA = − 126,522(− 161,221 to − 91,822) BD-FACSPresto vs FACSCount = − 38,708 (− 58,935 to − 18,482) and FACSPresto vs FACSCALIBUR = 0.791(− 11,908 to 13,491). Mean difference with Absolute CD4+ T-lymphocyte values obtained from the BD FACSPresto system correlated well with PIMA, FACSCount, and FACSCalibur method with R^2^ equal to 0.88, 0.92 and 0.968 respectively with P < 0.001 for all. The mean comparison between values obtained from BD FACSPresto with PIMA, FACSCount, and FACSCalibur using paired T test give P = 0.17, P = 0.5 and P = 0.6 respectively meaning that there is no significant differences between values obtained with BD FACSPresto and PIMA, FACSCount or FACSCalibur CD4 enumeration machines. Further analysis revealed close agreement between all the three instruments with no significant difference between the forth methods (P = 0.91).

**Conclusion:**

This BD-FACSPresto POC system is a simple, robust and reliable system for enumeration of absolute and percentage of CD4+ T-lymphocytes especially suitable for remote areas with limited resources. Having one BD-FACSPresto POC system easy to use, should reduce the cost and thus increase and improved access to CD4 testing for HIV infected patients in resource-constrained countries. BD-FACSPresto POC CD4 will enable reduction in patient time and improve the overall quality of ART service count and may improve test access in remote areas. This technology can allow for greater decentralization and wider access to CD4 testing and ART.

## Introduction

The number of people living with AIDS from the 2017 global epidemic of AIDS is about 36.9 million with 940,000 of AIDS related death. In Cameroon, the prevalence of HIV is about 3.7% in 2018 and about 510,000 people is living with HIV AIDS. Timely and appropriate initiation of antiretroviral treatment for HIV-positive subjects reduces morbidity and mortality associated with infections [1–5]. Moreover, eligibility for antiretroviral therapy for HIV/AIDS and monitoring progression of the disease commonly have been based on the number of CD4+ T lymphocytes in a patient’s venous blood [6–8]. In the context of the HIV and AIDS pandemic, the enumeration of CD4 antigen-positive T cells is used to determine the immune status of patients with immune deficiencies such as HIV/AIDS or suspected of becoming so. Cameroon has an HIV prevalence rate estimated at 3.7% with a strongly represented rural area [9, 10]. In this context, new tools for diagnosis and treatment are needed to improve care. In the context of good laboratory practice, the introduction of a new diagnostic tool requires an evaluation of the performance characteristics. In 2012, there was an emerging technology in point of care for the determination of CD4+ T lymphocytes with the aim to reduce problems of collection and transportation of samples and to reduce time delay for the results to patients. Previously, after analysis with conventional flow cytometry testing, the CD4 results may be available between two or more days after blood sampling, delaying the start of treatment for newly diagnosed patients and increasing the risk of loss-to-follow-up [11]. The introduction of point-of-care CD4+ cell counters in low income countries can improve access to quick and reliable CD4+ T-cell counts in HIV-positive patients because access to care and enabling initiation of treatment during a visit increase the efficiency and effectiveness for monitoring and staging of HIV patients [12, 13]. Absolute CD4+ cell count (AbsCD4) is a robust surrogate marker for immune competence in HIV-infected adults. However, percentage of CD4+ cells in the lymphocyte population (% CD4) has been considered a reliable surrogate marker for children less than 5 years of age, since the AbsCD4 count varies more than %CD4 due to the lymphocyte development cycle [14, 15]. In this study, the BD FACSPresto CD4 T cell counts were compared with BD FACSCalibur, BD facscount and Alere PIMA. In the national quality control program for external evaluation of CD4 coordinated by CIRCB reference laboratory, in collaboration with QASI of the Public Health Agency of Canada, each instruments passed in the year 2017 a quality control without any failure results. This is the first study done to compare the performance of instruments in our field context and this evaluation of the BD FACSPresto system comprised accuracy, precision and linearity. The CIRCB was commissioned to evaluate the BD FACSPresto ™ which is an automated system, CE IVD, designed for in vitro diagnostic use to perform direct enumeration of the absolute rate and percentage of CD4+ lymphocytes and the concentration of hemoglobin (Hb) in human blood. This is a prospective study to determine the relative bias between the BD FACSPresto ™ system and other CD4 counting devices present in Cameroon that perform an average of 5000 CD4+ T cell count tests per year. These are the BD FACSCalibur ™, reference device, BD FACSCount ™ and Alere PIMA. The choice of sites was based on the availability of the counting device and reagents.

## Materials and methods

Blood samples were obtained from patients undergoing routine CD4 monitoring at CIRCB, BIKOP, CASS de Nkolndongo and CMC of Nkolodom in Yaounde. All analyses were conducted in the medical analysis of each Unit. All specimens were processed within 24 h of collection. Blood was collected in K3-EDTA tubes and inverted several times to ensure proper mixing. For analysis on the FACSPresto analysis, a drop of blood from a Pasteur pipette was loaded onto the FACSPresto cartridge and capped and incubated at room temperature for 18 min; following incubation the cartridge was loaded onto the analyzer. For analysis on the PIMA, a drop of blood was loaded onto the cartridge and inserted into the analyzer. The TruCount method on the FACSCalibur was performed as the reference standard. In brief, 20 μl of BD Multitest fluorescent conjugated monoclonal antibodies, and 50 μl of whole blood were added to the TruCOUNT tube and vortexed for 5 s. The Multitest consists of CD3-FITC/CD8-PE/CD45-PerCP/CD4 APC reagent. The mixture was incubated for 15 min at room temperature in the dark before adding 450 μl of FACS™ lysing solution and incubating for an additional 15 min in the dark prior to acquisition on the FACSCalibur. Data were analyzed using the MultiSET™ software with automated gating and analysis. Analysis were conducted concurrently on the FACSCalibur, the FACSPresto, the FACSCount and the PIMA in a subset of specimens. Analyses were conducted on all 4 platforms for 265 specimens. Each operator had received adequate training on the use of reverse pipetting technique. All operators received training on the performance of the assay by the manufacturer. A total of 4 operators were involved in the evaluation. Maintenance and instrument calibration was performed according to the manufacturer’s guidelines and done prior to initiation of the evaluation. Internal quality control was monitored routinely and the labs participated in an external quality assurance program with the Cameroon National Quality Assurance Program for CD4 enumeration in collaboration with QASI in Canada and the lab that conducted the BD FACSCalibur subscribes also to the United Kingdom National External Quality Assurance Service (UK-NEQAS) for CD4 testing.

### Statistical methods

CD4+ T cell counts obtained from the FACSPresto device were compared to the FACSCalibur, FACSCount and PIMA. The amount of hemoglobin obtained from FACSPresto device were compared to the hematology device present in each center. Descriptive statistics was used to describe the data. Differences in parameters between the two groups were determined by Wilcoxon signed rank test and paired t-test. Passing–Bablok regression was used for the method correlation and correlation coefficients were determined. To determine the bias between the platforms, Bland–Altman analysis was done. The bias was defined as the mean difference between two methods. The limits of agreement were calculated as the mean ± 1.96 Standard Deviation (SD) of the differences of the results obtained. Confidence intervals for the bias and the limits of agreement were calculated. Pollock analysis was done to calculate the relative bias and the limits of agreement, which were defined as the mean ± 1.96 SD of the relative mean bias of paired measurements. The data was plotted with the y-axis representing the % difference relative to the absolute value (x-axis) of the comparator test. We determined the percentage similarity between a sample pair and defined it as the average between the methods divided by the comparator method multiplied by 100. The same analysis was done for comparing CD4% values on the FACSPresto, the FACSCount and the FACSCalibur. The coefficient of repeatability was calculated and was defined as the variation in triplicate measurements for 29 specimens, performed on the same instrument by a single technician under the same conditions. Coefficient of repeatability below 5% were considered optimal, coefficients between 5 and 10% were considered acceptable. Data was stratified into two groups based on CD4 count above or below 350 cells/μl and 500 cells/μl and CD4% above or below 25%. Agreement between the results obtained on the different platforms at CD4 counts below thresholds of 350 cells/μl and 500 cells/μl was determined using kappa statistics. We applied the Landis-Koch interpretation scale (kappa values of < 0.40 indicate poor agreement; > 0.40 and < 0.75 fair to good agreement and > 0.75 excellent agreement).

### Ethical review

The Cameroon National Ethics Committee approved the protocol prior to implementation with the number 2016/08/754/L/CNERH/SP. Blood samples were collected in the context of routine CD4 count testing for patients at CIRCB and other health facilities. Written informed consent is not required for receipt of routine services including CD4 testing performed at MOH facilities. Residual blood from routine testing was used for the analysis. No personally identifiable information was made available to the researchers. The institutional review boards waived the need for written informed consent.

## Results

Specimens collected from patients were tested on the FACSCalibur, FACSPresto, FACSCount and PIMA. The FACSPresto and FACSCalibur are present at CIRCB. PIMA Alere and BD FACSCount are present at BIKOP and CMC of Nkolondom respectively. CD4 devices used in this work are representative in Cameroon according to the number of tests per year. Each CD4 device is doing per year more than 5000 CD4 enumeration tests (personal data).

**Fig. 1:**
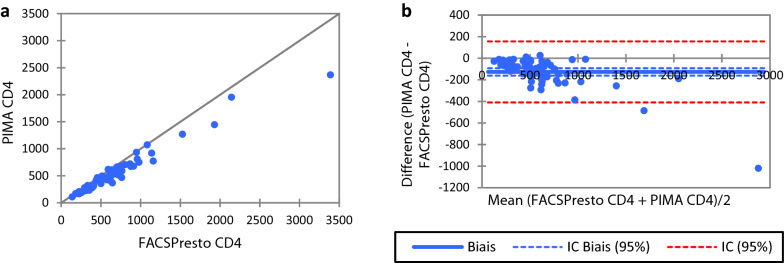
Comparison between FACSPresto and PIMA. Passing-Bablok regression plot comparison of **a** absolute CD4 count values obtained from FACSPresto with the PIMA as reference standard. The solid line represents the regression line and dashed line the 95% CI. Pollock plots indicating  %mean bias between **b** absolute CD4 counts values obtained on FACSPresto compared with those obtained on the FACSCount. The solid line represents the mean bias, the dashed line represents mean bias ± 1.96SD

**Fig. 2:**
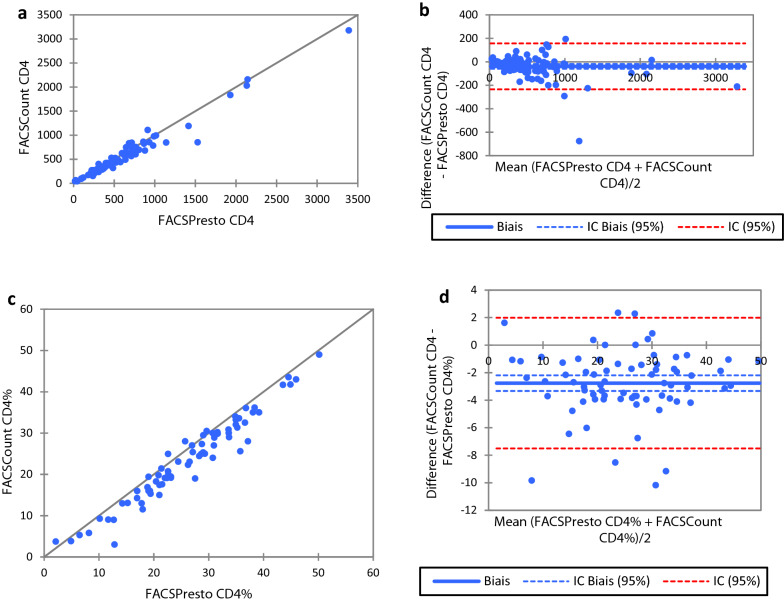
Comparison between FACSPresto and FACSCount. Passing-Bablok regression plot comparison of **a** absolute CD4 count and **c** CD4% values obtained from FACSPresto with the FACSCount as reference standard. The solid line represents the regression line and dashed line the 95% CI. Pollockplots indicating %mean bias between **b** absolute CD4 count and **d** CD4% values obtained on FACSPresto compared with those obtained on theFACSCount. The solid line represents the mean bias, the dashed line represents mean bias ± 1.96SD

**Fig. 3:**
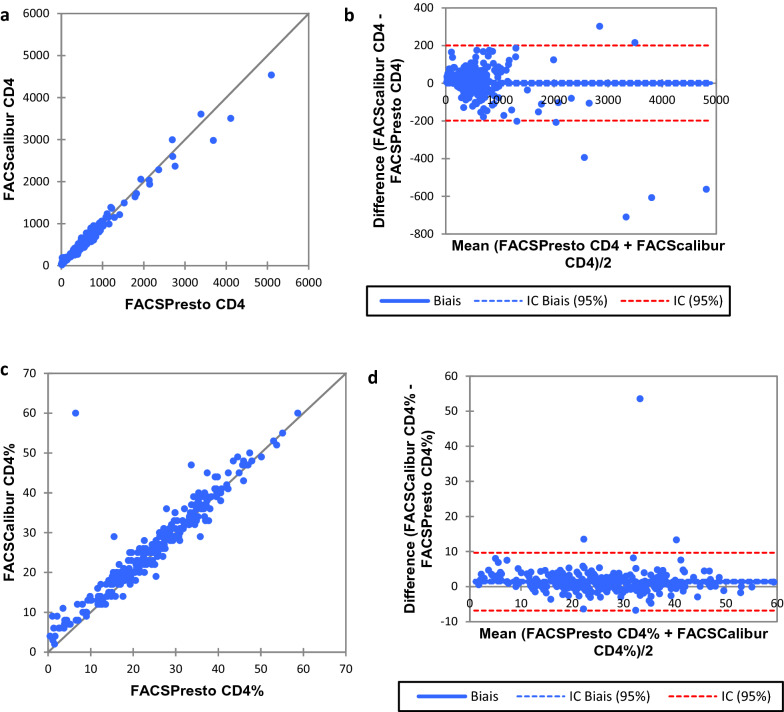
Comparison between FACSPresto and FACSCalibur. Passing-Bablok regression plot comparison of **s** absolute CD4 count and **c** CD4% values obtained from FACSPresto with the FACSCalibur as reference standard. The solid line represents the regression line and dashed line the 95% CI. Pollock plots indicating %mean bias between **b** absolute CD4 count and **d** CD4% values obtained on FACSPresto compared with those obtained on the FACSCalibur. The solid line represents the mean bias, the dashed line represents mean bias ± 1.96SD

69 samples were tested on PIMA and FACSPresto (Fig. 1) with the ration of 0.28 between men and woman, 96 samples were tested on FACSCount and FACSPresto (Fig. 2) with the ratio of 0.32 between men and woman and 245 samples were tested on FACSCalibur and FACSPresto (Fig. 3) with the ratio of 0.26 between men and woman.

A total of 245 patients were enrolled in this technical accuracy evaluation. During the study, we processed the EQA sample from QASI and the values obtained by different devices were proficient.

The BD FACSPresto evaluated had CE-IVD and WHO-PQ (pre-qualification) approval. Approximately 75% of enrolled patients were female while 33% and 53% of patients had a CD4+ T cell count above 350 cells/ul, and 500 cells/µl as measured by the BD FACSCalibur. The mean and median ages of enrolled participants were 36 Table 1.

**Table 1:** Characteristics of study participants

	Men	Female	Children	Total
Participants	46	158	41	245
Mean age	39.41	40.86	9	35.46
T CD4 absolute value				
Mean FACSCalibur	435	494	1236	588
Mean FACSPresto	436	480	1286	589
Mean FACSCount	433	494	925	484
Mean PIMA	423	502	1248	507
% T CD4				
Mean %FACSCalibur	23.24	25.84	32.93	26
Mean %FACSPresto	22.32	24.5	30.73	25
Mean %FACSCount	21.32	25.07	23.75	23

Samples tested using the BD FACSCalibur had a median of 609 cells/ul (range: 5–1776 cells/ul), compared with a median of 608 cells/ul (range: 6–1607 cells/ul) on the BD FACSCount compared with a median of 563 cells/ul (range: 6–1635 cells/ul) on the BD FACSPresto and a median of 540 cells/µl on the Alere PIMA. The patient distributions by technology and CD4+ T cell threshold can be found in Table 1.

The BD FACSCalibur and BD FACSPresto classified 33% and 30% of enrolled patients as above 350 cells/ul, respectively. The Alere PIMA and BD FACSPresto classified 30% and 25% of enrolled patients as above 350 cells/µl respectively.

The BD FACSCount and BD FACSPresto classified 39% and 37% of enrolled patients as above 350 cells/µl respectively.

## Discussion

The Spearman correlation coefficient is good for all three instruments compared to BD FACSPresto and the P value is less than 0.05 (Table 2). An important bottleneck for the extension of patient care services and ARV treatment in Cameroon is the access to diagnosis for the identification and classification of patients faced with difficulties such as stock-outs, equipment maintenance. The introduction of rapid testing for HIV in the last decade has facilitated people’s access to HIV testing services, followed by care and treatment services. Access to local CD4 tests has been made possible thanks to the PIMA devices of the ALERE group [16, 17] but remains insufficient and limited because a good portion of the population aged 0 to 5 years does not have access to this test directly because it requires using a hematology device to get the percentage.

**Table 2:** Coefficient of Spearman and p-value between BD FACSPresto and other instruments

	Spearman's rank correlation coefficient	P-value
Variables	BD FACSPresto™ CD4	BD FACSPresto™ CD4
BD FACSCalibur™ CD4	*0.981*	*0.0001*
BD FACSPresto™ CD4	*1*	0
BD FACSCount™ CD4	*0.974*	< *0.0001*
PIMA CD4	*0.963*	< *0.0001*

The values in italic are different from 0 to a significant level alpha = 0.05

The follow-up of children under 5 years benefits from measuring the percentage of CD4+ T cells with BD FACSPresto ™ while the PIMA does not give this value which is important in improving the management of these patients. The BD FACSPresto ™ has the advantage of providing in absolute value and percentage CD4+ T cells as well as the hemoglobin level in addition to the fact that it can make between 60 and 80 samples per day.

The internal quality control does not require additional reagents or cartridges, and it reads the external quality control samples provided by QASI and UKNEQAS without any problem. It is easy to use because everything is shown in video image on the touch screen of the device. It can be used in rural areas where electrical problems are lacking because it has a battery that has a battery life of 6 h. It does not require accessory instruments, computers, pipettes or consumables (other than lancets, swabs and gloves) for sampling. Cartridges can be stored at room temperature, as the cold chain is no longer available. Necessary thereby reducing the cost of transport and storage. The results are stored by the instrument up to 12,000. The BD FACSPresto ™ device has features that are suitable for use in districts and rural health centers improving access to care and reducing mortality and morbidity [18]. It will also reduce queues while reducing patient visits to health facilities as patients with immunodeficiency are prioritized and expected to reduce energy costs. Similar studies have been done in Kenya and many other countries using whole blood with EDTA anticoagulant and capillary blood to see the correlation [19–23]. The slight discrepancy between the individual results of BD FACSPresto ™ and other devices in this study was similar to those seen and accepted in other comparisons of conventional CD4 technologies (Additional file 1). The discrepancy is likely to be due to an error caused by variations in blood collection or pipetting.

## Conclusion

Given the low sensitivity and high upward misclassification rates, it would be worthwhile to understand the performance of the BD FACSPresto in sicker populations of HIV-positive patients. Given the need to expand access to CD4+ T cell testing for pre-ART patient management and replace old or broken conventional technologies, the BD FACSPresto performs well in the laboratory setting and could be considered in rational deployment of future POC CD4+ T cell technologies.

### Study limits

The study was conducted at CIRCB where the staff is qualified and the blood collected in EDTA tubes. Tests on capillary blood have not been done and it would be necessary to perform them in rural areas where the conditions are very different in terms of personnel and workload. Information on the cost of the test and the cost of the device was not provided.

## Supplementary information

**Additional file 1.** Summary of various CD4+ T cells enumeration techniques.

## Data Availability

Not applicable.

## References

[1] Meyers K, Qian H, Wu Y, Lao Y, Chen Q (2015). Early initiation of ARV during pregnancy to move towards virtual elimination of mother-to-child-transmission of HIV-1 in Yunnan, China. PLoS ONE.

[2] van Griensven J, Thai S (2011). Predictors of immune recovery and the association with late mortality while on antiretroviral treatment in Cambodia. Trans R Soc Trop Med Hyg.

[3] Collaborators GH (2016). Estimates of global, regional, and national incidence, prevalence, and mortality of HIV, 1980–2015: the Global Burden of Disease Study 2015. Lancet HIV.

[4] Cohen MS, Chen YQ, McCauley M, Gamble T, Hosseinipour MC (2011). Prevention of HIV-1 infection with early antiretroviral therapy. N Engl J Med.

[5] Tanser F, Barnighausen T, Grapsa E, Zaidi J, Newell ML (2013). High coverage of ART associated with decline in risk of HIV acquisition in rural KwaZulu-Natal, South Africa. Science.

[6] Peeling RW, Sollis KA, Glover S, Crowe SM, Landay AL (2015). CD4 enumeration technologies: a systematic review of test performance for determining eligibility for antiretroviral therapy. PLoS ONE.

[7] Sterling TR, Chaisson RE, Moore RD (2001). HIV-1 RNA, CD4 T-lymphocytes, and clinical response to highly active antiretroviral therapy. AIDS.

[8] Giorgi JV (1993). Characterization of T lymphocyte subset alterations by flow cytometry in HIV disease. Ann N Y Acad Sci.

[9] Sama CB, Feteh VF, Tindong M, Tanyi JT, Bihle NM (2017). Prevalence of maternal HIV infection and knowledge on mother-to-child transmission of HIV and its prevention among antenatal care attendees in a rural area in northwest Cameroon. PLoS ONE.

[10] De Beaudrap P, Beninguisse G, Pasquier E, Tchoumkeu A, Touko A (2017). Prevalence of HIV infection among people with disabilities: a population-based observational study in Yaounde, Cameroon (HandiVIH). Lancet HIV.

[11] Boyle DS, Hawkins KR, Steele MS, Singhal M, Cheng X (2012). Emerging technologies for point-of-care CD4 T-lymphocyte counting. Trends Biotechnol.

[12] Stevens WS, Gous NM, MacLeod WB, Long LC, Variava E (2017). Multidisciplinary Point-of-Care testing in South African primary health care clinics accelerates HIV ART initiation but does not alter retention in care. J Acquir Immune Defic Syndr.

[13] Vojnov L, Markby J, Boeke C, Harris L, Ford N (2016). POC CD4 testing improves linkage to HIV care and timeliness of ART initiation in a public health approach: a systematic review and meta-analysis. PLoS ONE.

[14] Karanth SS, Rau NR, Gupta A, Kamath A, Shanbhogue V (2014). Utility of total lymphocyte count as a surrogate for absolute CD4 count in the adult Indian HIV population: a prospective study. Avicenna J Med.

[15] Ghebremichael M, Michael H, Tubbs J, Paintsil E (2019). Comparing the diagnostics accuracy of CD4+ T-lymphocyte count and percent as a surrogate markers of pediatric HIV disease. J Math Stat.

[16] Wade D, Daneau G, Aboud S, Vercauteren GH, Urassa WS (2014). WHO multicenter evaluation of FACSCount CD4 and Pima CD4 T-cell count systems: instrument performance and misclassification of HIV-infected patients. J Acquir Immune Defic Syndr.

[17] Morawski BM, Meya DB, Boulware DR (2013). Accuracy of pima point-of-care CD4 analyzer in routine use in public health clinics in Uganda. J Acquir Immune Defic Syndr.

[18] Bekolo CE, Webster J, Batenganya M, Sume GE, Kollo B (2013). Trends in mortality and loss to follow-up in HIV care at the Nkongsamba Regional hospital, Cameroon. BMC Res Notes.

[19] Coetzee LM, Moodley K, Glencross DK (2016). Performance evaluation of the Becton Dickinson FACSPresto near-patient CD4 instrument in a laboratory and typical field clinic setting in South Africa. PLoS ONE.

[20] Makadzange AT, Bogezi C, Boyd K, Gumbo A, Mukura D (2016). Evaluation of the FACSPresto, a new point of care device for the enumeration of CD4% and absolute CD4+ T cell counts in HIV infection. PLoS ONE.

[21] Daneau G, Aboud S, Prat I, Urassa W, Kestens L (2017). Performance of FACSPresto point-of-care instrument for CD4-T cell enumeration in human immunodeficiency virus (HIV)-infected patients attending care and treatment clinics in Belgium and Tanzania. PLoS ONE.

[22] Bwana P, Vojnov L, Adhiambo M, Akinyi C, Mwende J (2016). Correction: the BD FACSPresto point of care CD4 Test accurately enumerates CD4+ T cell counts. PLoS ONE.

[23] Angira F, Akoth B, Omolo P, Opollo V, Bornheimer S (2016). Clinical evaluation of the BD FACSPresto near-patient CD4 counter in Kenya. PLoS ONE.

