# Supplementary material for: Performance of the BD FACSPresto near to patient analyzer in comparison with representative conventional CD4 instruments in Cameroon
Source: AIDS Res Ther. 2020 Aug 17;17:53. doi: 10.1186/s12981-020-00309-9 (PMC7429678; doi:10.1186/s12981-020-00309-9)
Supplement: Supplementary file 1 — Additional file 1. Summary of various CD4+ T cells enumeration techniques. [file 12981_2020_309_MOESM1_ESM.docx]

Additional file 1

| **S(1): Summary of various CD4+ T cells enumeration techniques** | | | | |
| --- | --- | --- | --- | --- |
| Parameter | Dedicated technology based assay | | | |
|  | FACSCalibur | FACSCount | FACSPresto | PIMA |
| Manufacturer | Becton Dickinson (CA, USA) | Becton Dickinson (CA, USA) | Becton Dickinson (CA, USA) | Alere Medical Pvt. Ltd, USA |
| Plateform | TruCount | Dedicated CD4/CD4% counter | Dedicated CD4 Count | Dedicated CD4 Count |
| Monoclonal antibodies used | Multitest CD3/CD8/CD45/CD4 | Anti-CD4 &anti CD3 | Anti-CD3, anti-CD4 | Anti-CD3, anti-CD4 |
| specimen volume (µL) | 50 | 50 | 1-2 drops of venus or capillary whole blood | 1-2 drops of venus or capillary whole blood |
| Gating strategy | CD45/SSC | CD3/SSC | CD4/CD3/CD45RA/CD14 | CD3 |
| Robustness |  |  | ROBUST | ROBUST |
| time taken to perform a test | 30 | 40 | 25 | 20 |
| estimated cost of the instrument | 75000 | 30000 | 8000 | 12000 |
| cost of the test in $US | 25 | 20 | 12 | 12 |
| number of test performed per day | 200-250 | 50-60 | 60 | 24 |
| Technical skills required | REQUIRE SOME SKILL | REQUIRE SOME SKILLS | EASY TO USE | EASY TO USE |
| Adaptability in Africa | 2 | 2 | 4 | 3 |
| type of test | abs and percentage of total CD3, CD3CD4, CD3CD8 and the ratio CD4/CD8 | CD3/CD4 | abs and percentage of CD4 and the total hemoglobin | abs CD4 |
| Structure | CIRCB | CMA NKOLODOM | CIRCB | CASS, CMA BIKOP |
| abs=absolute | |  |  |  |
| CIRCB: Chantal BIYA International Reference Center | | | |  |
| CMA: Centre Medical d'Arrondissement | | |  |  |
| CASS: Centre d'Animation Social et Sanitaire | | |  |  |
